# Difference in Endocytosis Pathways Used by Differentiated Versus Nondifferentiated Epithelial Caco-2 Cells to Internalize Nanosized Particles

**DOI:** 10.1021/acs.molpharmaceut.4c00333

**Published:** 2024-06-12

**Authors:** Azzah Bannunah, Robert Cavanagh, Saif Shubber, Driton Vllasaliu, Snow Stolnik

**Affiliations:** † School of Pharmacy, University of Nottingham, University Park, Nottingham NG7 2RD, U.K.; ‡ School of Cancer & Pharmaceutical Sciences, Faculty of Life Sciences & Medicine, King’s College London, Franklin-Wilkins Building, 150 Stamford Street, London SE1 9NH, U.K.

**Keywords:** endocytosis, inhibitors, polarized and differentiated cells, epithelial cell uptake

## Abstract

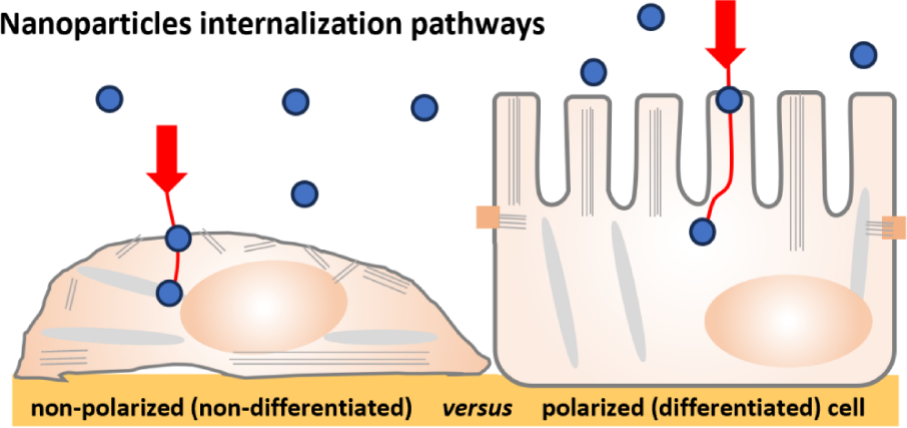

Understanding the internalization of nanosized particles by mucosal epithelial cells is essential in a number of areas including viral entry at mucosal surfaces, nanoplastic pollution, as well as design and development of nanotechnology-type medicines. Here, we report our comparative study on pathways of cellular internalization in epithelial Caco-2 cells cultured in vitro as either a polarized, differentiated cell layer or as nonpolarized, nondifferentiated cells. The study reveals a number of differences in the extent that endocytic processes are used by cells, depending on their differentiation status and the nature of applied nanoparticles. In *polarized cells*, actin-driven and dynamin-independent macropinocytosis plays a prominent role in the internalization of both positively and negatively charged nanoparticles, contrary to its modest contribution in nonpolarized cells. Clathrin-mediated cellular entry plays a prominent role in the endocytosis of positive nanoparticles and cholesterol inhibition in negative nanoparticles. However, in *nonpolarized cells,* dynamin-dependent endocytosis is a major pathway in the internalization of both positive and negative nanoparticles. Cholesterol depletion affects both nonpolarized and polarized cells’ internalization of positive and negative nanoparticles, which, in addition to the effect of cholesterol-binding inhibitors on the internalization of negative nanoparticles, indicates the importance of membrane cholesterol in endocytosis. The data collectively provide a new contribution to understanding endocytic pathways in epithelial cells, particularly pointing to the importance of the cell differentiation stage and the nature of the cargo.

## Introduction

Understanding the mechanisms of internalization into and transport of nanosized materials across mucosal epithelial cell layer is essential in a number of areas. These include, for example, viral entry at mucosal surfaces and consequent viral infections, such as SARS-CoV,^1^ nanoplastic pollution, as well as a development of nanotechnology-type medicines.^2,3^ Epithelial cells in the mucosal barrier layer have evolved a characteristic polarization of cellular structures. For instance, in Madin-Darby canine kidney (MDCK) cells, the most extensively studied polarized cell model, the microtubules in nonpolarized cells are nucleated by the centrosome, whereas in polarized cells, the microtubular network is reorganized parallel to the apico-basal polarity axis with their minus ends underneath the apical surface.^4^ Additionally, in polarized cells, a horizontal network of actin filaments is present underlying the apical surface, from which actin organizes to form apical microvilli.^5^ The polarization further includes the presence of apical and basolateral membrane domains, separated by tight junctions, with specific protein and lipid composition.^6^ It should be noted, however, that the membrane structures thought to play a key role in the apical to basolateral distinctiveness, namely, lipid rafts formed from glycosphingolipids and cholesterol, have also been shown to be present in nonpolarized cells. In polarized cells, lipid rafts are present as a distinctive clustering to facilitate the sorting and segregation of specific functional membrane proteins, whereas in nonpolarized cells, rafts are distributed randomly on the cell surface.^7,8^

Previous evidence indicates that in polarized, differentiated epithelium, endocytic mechanisms may be membrane region specific, and distinct mechanisms may operate at the apical and basolateral membranes.^9,10^ This has ramifications for studying the internalization of nanosized delivery system. The majority of the current knowledge on internalization mechanisms of nanosized particles for drug delivery arises from studies conducted in cells grown following standard procedures, usually as subconfluent, short-term culture on tissue culture plasticware, i.e., nonpolarized cell culture.^11,12^ Studies on the mechanisms of cellular internalization into, and transport across, polarized cell layers are of increased complexity and time requirements as cells are grown in specific in vitro culture conditions, typically on semipermeable membranes, until acquiring properties representative of epithelium, including the formation of tight junctions and actin organization.

The Caco-2 epithelial model is the most widely utilized model for the study of oral drug delivery and is the industry standard for drug permeability studies. Nonpolarized, undifferentiated Caco-2 cells are reported to resemble the tumorigenic phenotype of colon carcinoma cells, whereas upon differentiation into polarized cell layers, Caco-2 cells obtain a phenotype similar to enterocytes in mucosal tissue with respect to morphology and functionality.^13^

Here, we employ a panel of pharmacological inhibitors of endocytosis to compare cellular internalization pathways of nanosized (approximately 100 nm in diameter) particles by nonpolarized versus polarized, differentiated cell layers in Caco-2 cell culture. The study is conducted in the absence of any specific targeting, i.e., presence of specific ligands on the surface of nanoparticles designed to interact with specific plasma membrane receptors at the cell surface. We employed model polystyrene nanoparticles with negative and positive surface charge to reflect the widely discussed effects of surface charge on cellular internalization of nanoparticles,^14^ with the latter system modeling often present positive charge of nucleic acids delivery systems, formulated either as polyplexes or lipoplexes (polymeric or lipid-based complexes, respectively).^15^ As this work addresses a fundamental question of whether endocytosis is different in nonpolarized and polarized cells, experiments were conducted in a basic cell culture medium as the presence of different serum components, and their differing adsorption onto negative and positive nanoparticle surface, would add another layer of complexity of effect of “protein corona” to data interpretation.

## Materials and Methods

Caco-2 cells (human intestinal adenocarcinoma) were obtained from the European Collection of Cell Cultures (ECACC) and were used between passages 25 and 40. Eagle’s minimum essential medium (EMEM), 2.5% trypsin/EDTA, antibiotic/antimycotic solution, fetal bovine serum (FBS, non-USA origin), l-glutamine, and nonessential amino acids (NEAA) were purchased from Sigma-Aldrich (UK). Hank’s balanced salt solution (HBSS), Triton X-100, and 4-(2-hydroxyethyI)-1-piperazineethanesulfonic acid (HEPES) were also obtained from Sigma-Aldrich. Phosphate-buffered saline (PBS) was purchased from Oxoid Ltd. (UK). Nocodazole, 5-(N-ethyl-N-isopropyi)-amiloride (EIPA), methyl-ß-cyclodextrin (MβC), nystatin, filipin, and chlorpromazine were obtained from Sigma-Aldrich (UK). Genistein and dynasore were purchased from Tocris Bioscience and Calbiochem (UK). All other chemicals were obtained from Sigma-Aldrich.

## Cell Culture

Caco-2 cells were routinely cultured in 75 cm^2^ tissue culture flasks at 5% CO_2_, 95% relative humidity, and 37 °C using Eagle’s minimum essential medium (EMEM) supplemented with fetal bovine serum (FBS), nonessential amino acids, l-glutamine, and antibiotics/antimycotics. Caco-2 cells were seeded on Transwell permeable filter supports (12 mm diameter; 0.4 μm pore size membrane) (Corning) at 1.25 × 10^5^ cells per well/insert. The cell layer formation and its integrity were assessed by periodical measurements of the transepithelial electrical resistance (TEER), as illustrated in Figure S1. Cell layers were typically used at days 21–23 post seeding.

To form nonpolarized Caco-2 cell culture, cells were seeded at 10^5^ per cm^2^ and cultured in a sterile 12-well polystyrene plate (Corning) for 2 days before used in further experiments.

## Nanoparticles and Cell Toxicity

Orange aminated polystyrene nanoparticle (100 nm) suspension was obtained from Sigma-Aldrich. Fluoresbrite carboxy-modified nanoparticle (100 nm) suspension was purchased from Polysciences Ltd. (Germany). Physicochemical characterization of nanoparticles used in the study is presented in Figure S2 and summarized in Table S1. Mass of the nanoparticles applied to cells was calculated from the suppliers’ specifications (from %w/v). From serial dilutions of the nanoparticle suspensions, calibration curves were constructed from fluorescence readings (TECAN Infinite M200 plate reader), and these were used to assess the mass of nanoparticles associated with cells. The cytotoxicity of the nanoparticles used in the study is shown in Figure S3 a,b, investigated using the MTS (CellTiter 96 AQueous MTS) and LDH release assays (Sigma TOX7, UK) to assess cellular metabolic activity and plasma membrane integrity, respectively. Assays were performed as per manufacturers’ instructions. For Caco-2 cells cultured on Transwell permeable membrane, TEER measurements were taken as measure of cell toxicity, as shown in Supporting Information Figure S3 b,c.

## Transmission Electron Microscopy

Transwell membrane cultured confluent Caco-2 cell layers were fixed with 4% paraformaldehyde and then washed with 0.1 M phosphate/cacodylate buffer. Cells were post fixed with 1% aqueous osmium tetroxide for 30 min, followed by extensive washing with distilled water. Samples were then dehydrated in a graded ethanol series by washing the cells for 2 × 5 min with 50% ethanol, followed by 70% and 90% ethanol and then finally with 3 × 10 min washes with pure ethanol. Finally, two 5-min washes were performed with 100% dried acetone. Cell samples were then infiltrated with resin for a duration of 30 min in 1:3 resin:acetone mixture, 1 h in 1:1 resin:acetone mixture, and 3 × 1 h incubations in pure resin. Samples were embedded in the resin and left in the embedding oven for 48 h at 60 °C. Once the resin block polymerized, the substrate was removed from the cells by immersing the block in liquid nitrogen and snapping the substrate from the block. The block was then embedded in a microcentrifuge tube containing fresh resin and was polymerized as before, enclosing the cell layer in a resin block. The disc was cut in half to form 2 semicircles, which were stuck onto the top of a blank resin block (using Araldite epoxy resin). The Araldite was left to set for 1 h. Ultrathin sections were cut using a cryo-ultramicrotome (Leica EM, UC6/FC6, Milton Keynes, UK) and viewed by TEM (Jeol JEM 1010 Electron Microscope, Japan).

## Immunostaining

Confluent, filter-cultured cell layers were washed and fixed in 4% paraformaldehyde diluted in PBS for 10 min at room temperature. Cells were then washed with PBS and permeabilized by incubating with Triton X-100 (0.2% v/v in PBS) for 10 min, washed with PBS again, and incubated with blocking solution (1% BSA:PBS) for 1 h. In the following step, cells were stained for F-actin, zonula occludens (ZO-1), or caveolin. To stain for F-actin, 0.17 μM Alexa Fluor-546 phalloidin in 1% BSA:PBS solution was applied to cells and incubated for 20 min, as per the manufacturer’s instructions (Thermo Fisher Scientific). Nuclei were stained using 25 μg/mL Hoechst 33 342 diluted in a PBS solution for 10 min. For ZO-1 tight junction staining, mouse anti-human ZO-1 (primary) antibody (Thermo Fisher Scientific) was applied at 10 μg/mL diluted in 1% BSA:PBS solution and incubated with cells for 45 min. Primary antibody solution was then removed, and cells were washed five times with PBS. Alexa fluor 594-labeled goat, anti-mouse (secondary) antibody (Thermo Fisher Scientific), diluted according to manufacturer’s instructions in 1% BSA:PBS solution was then applied to the cells for 30 min. Caveolin immunostaining was performed with primary rabbit antibody, anti-human caveolin1 H-97 (Santa Cruz Biotechnology, Inc.) diluted 1:50 with 1% BSA:PBS solution and incubated with the cell layers for 30 min. For clathrin immunostaining, the cell layers were treated with rabbit anticlathrin primary antibody (Abcam, UK) diluted 1:200 in 1% BSA:PBS solution. For both caveolin and clathrin staining, the primary antibody was removed from the cell layers, and the cell layers were washed five times with PBS. For clathrin staining, the secondary antibody used was goat, anti-rabbit IgG-FITC antibody, and for caveolin staining, goat, antirabbit IgG-rhodamine antibody was diluted 1:100 in 1% BSA:PBS solution and incubated with cell layers for 30 min. The control cell layers were incubated with the secondary antibody only. Alternative secondary antibody staining for caveolin was performed with (goat, antirabbit IgG-FITC), as shown in Figure S4. Following immunostaining of ZO-1, caveolin, or clathrin proteins, secondary antibodies were removed, and cells were washed extensively with PBS and counterstained with 100 μg/mL Hoechst 33 342 for 5 min to visualize cell nuclei. Transwell membrane filters containing stained cell monolayers were excised from inserts using a scalpel and mounted onto glass slides using 1% DABCO (1,4-diazabicyclo[2.2.2]octane) diluted in 9:1 glycerol:PBS, and glass coverslips applied. Confocal imaging was then performed using a Leica TCS SP2 system mounted on a Leica DMIRE2 inverted microscope.

## Inhibition of Cellular Internalization Pathways

Chemical inhibitors were applied to Caco-2 cells grown either on standard tissue culture plasticware or on permeable membrane inserts to investigate the endocytosis pathways exploited by nanoparticles. Selected inhibitors were optimized according to dose-limiting cytotoxicity (Figure S5 and Table S2) and include dynasore, genistein, 5-(N-ethyl-*N*-isopropyl)-amiloride (EIPA), chlorpromazine, filipin, nystatin, MβC, and nocodazole. An illustration summarizing the effects of selected inhibitors on endocytosis routes is presented in Schematic 1. Initial validation of chemical inhibitor action was investigated against the internalization of “classical” ligands, including cholera-β-toxin (CβT) and transferrin (TF), and is shown in Figures S7 and S8.

**Scheme 1:**
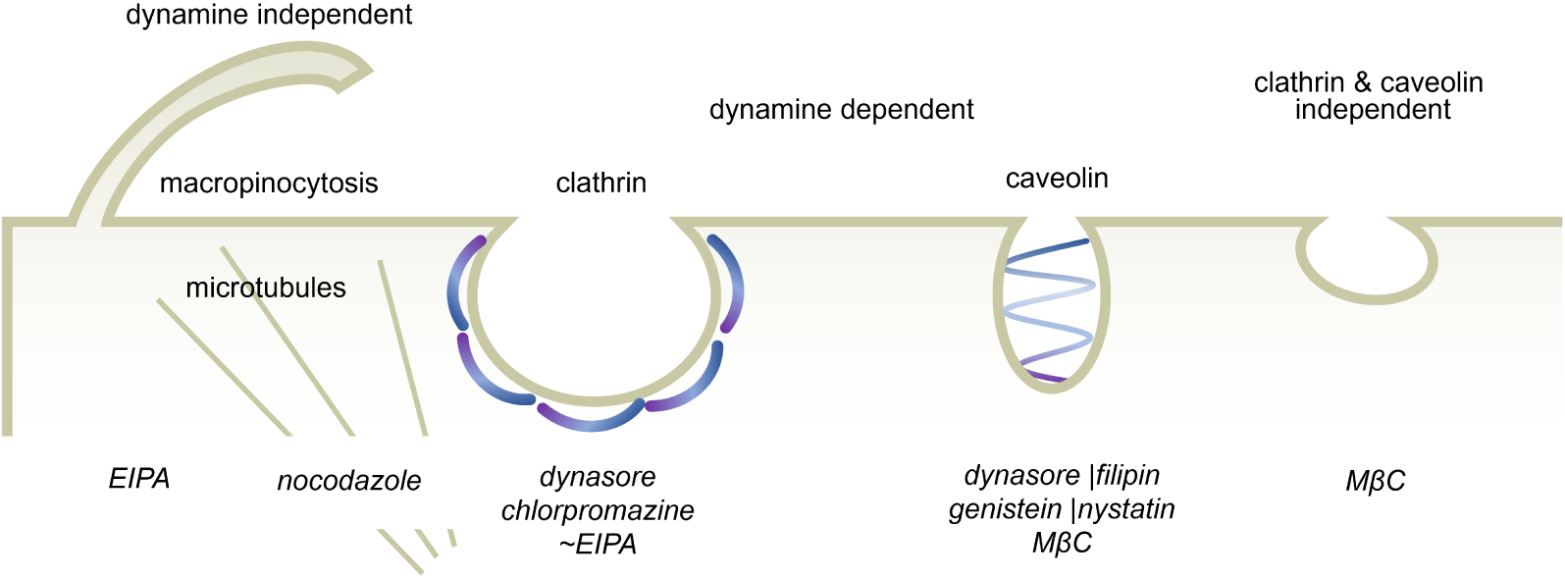
Illustration of the Effects of Pharmacological Inhibitors (Indicated in Italics) Used in This Study on Endocytosis Processes

Inhibitor compounds were dissolved in HBSS containing 20 mM HEPES and applied to cells for 30 min at 37 °C; i.e., applied to a well for standard tissue culture plate cultured cells or to the apical side of inserts for permeable membrane cultured cells. Following this inhibitor preincubation, solutions were removed, and nanoparticle (50 μg/mL) suspensions, containing the relevant inhibitor, were applied to cells in 20 mM HEPES:HBSS buffer. Control experiments were performed in the absence of inhibitor and at the corresponding time points, to ascertain baseline levels of nanoparticles internalization by cells. Nanoparticles were incubated with cells for 120 or 180 min, after which suspensions were removed, and cells were washed extensively 3 times with PBS, adopting a protocol described and shown previously to optimally remove nanoparticles adhered on the cell surface.^16^ Cells were subsequently lysed with 0.2% (v/v) Triton X-100 for 10 min. The resulting mixtures were collected and centrifuged at 13 000 rpm for 5 min to remove debris. Fluorescence of the supernatant samples was measured on an TECAN Infinite M200 plate reader. Detection of 100 nm aminated nanoparticles was performed at λ_ex_/λ_em_ 481/644 nm wavelengths and 100 nm carboxylated latex nanoparticles at λ_ex_/λ_em_ 529/590 nm.

All data are presented as mean ± standard deviation with a minimum of three repeats. Student’s *t* tests were performed for comparisons of two group mean values, while one-way analysis of variance (ANOVA) followed by Bonferroni posthoc test was applied for the comparison of three or more group mean values. A *p* value of <0.05 was considered statistically significant. ****, ***, **, and * display *p* < 0.0001, *p* < 0.001, *p* < 0.01, and *p* < 0.05, respectively, whereas “ns” indicates nonsignificant.

## Results and Discussion

### Cell Culture and Nanoparticle Internalization

**Figure 1:**
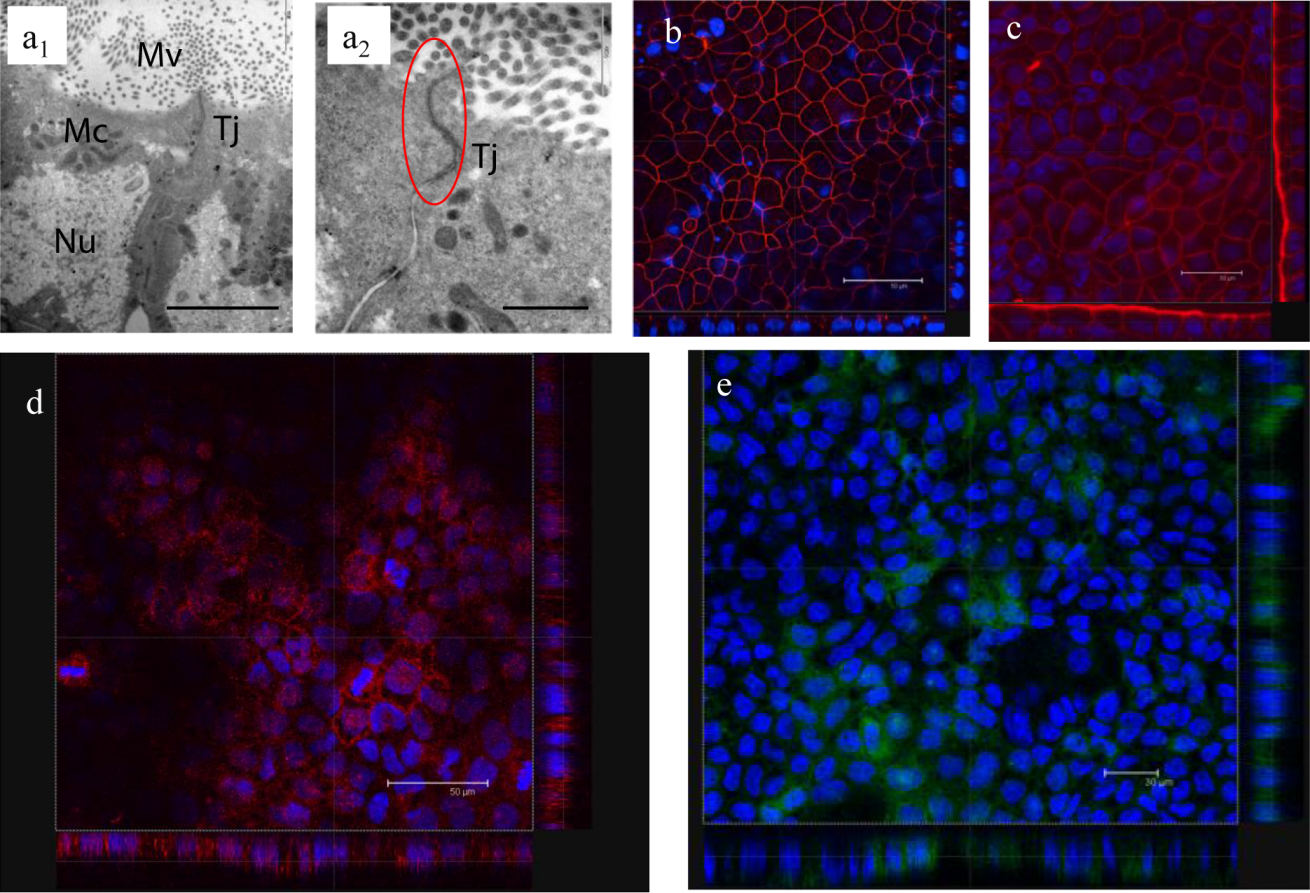
Characterization of semipermeable membrane (Transwell) cultured Caco-2 cells. (a) Transmission electron microscopy (TEM) micrographs depicting adjacent Caco-2 cells connected by a tight junction (Tj) (image a_1_) and a magnified region (in image a_2_) depicting a tight junctional boundary (Tj circled in red), labeled cell nucleus (Nu), mitochondria (Mc), and microvilli (Mv). Scale bars represent 2 μm (image a_1_) and 1 μm (image a_2_). (b) Overlay confocal microscopy image of Caco-2 cell layer showing DAPI-labeled cell nuclei (blue) and ZO-1 tight junction protein distribution (red Alexa fluor594-labeled goat, anti-mouse IgG, and mouse, anti-human ZO-1), scale bar 50 μm, edge panels show x-z and y–z planes and ZO-1 red staining above the level of cell nuclei. (c) F-actin staining in polarized cell layer; DAPI (blue) staining of nuclei and phalloidin (Alexa Fluor-546 phalloidin) (red) staining of F-actin, edge panels show x-z and y–z planes and illustrate red phalloidin staining above the level of cell nuclei; scale bar 30 μm. (d) Expression of caveolin-1 in differentiated Caco-2 cells by immunostaining; cell layers were permeabilized (as described in Materials and Methods) and treated with antihuman caveolin-1 H-97 antibody, followed by goat, anti-rabbit IgG-rhodamine (red) and Hoechst-labeled cell nuclei (blue); scale bar 50 μm; alternative staining with anti-rabbit IgG-FITC secondary antibody shown in Figure S4. (e) Expression of clathrin; cells treated with rabbit anti-clathrin primary antibody and goat, anti-rabbit IgG-FITC secondary antibody (green) and Hoechst-labeled cell nuclei; scale bar 30 μm; edge panels in images b–e show x-z and y*-*z planes.

Initially, we assessed the structural and morphological characteristics of differentiated, polarized Caco-2 layers cultured on semipermeable membrane filters (Figure 1). Images illustrate the classical feature of such cultured cells, namely, the formation of tight junctions, as indicated morphologically by transmission electron microscopy (TEM) (Figure 1a) and the presence and distribution of ZO-1 protein at cellular boundaries within cell layers observed via confocal microscopy (Figure 1b). Confocal microscopy in x-z and y-z planes further illustrates tight junctions’ position above the cell nuclei level, indicative of well-formed epithelial layer (Figure 1b). Corresponding development of TEER is illustrated in Figure S1. Image c illustrates actin staining of a differentiated cell layer, with x-z and y–z planes illustrating the presence of apical and lateral actin cytoskeleton, showing “accumulation” of actin at the apical side of polarized cells.^5^ Images d and e illustrate the expression of caveolin and clathrin proteins by the cell layers, respectively, as illustrated by relevant immunostaining.

**Figure 2:**
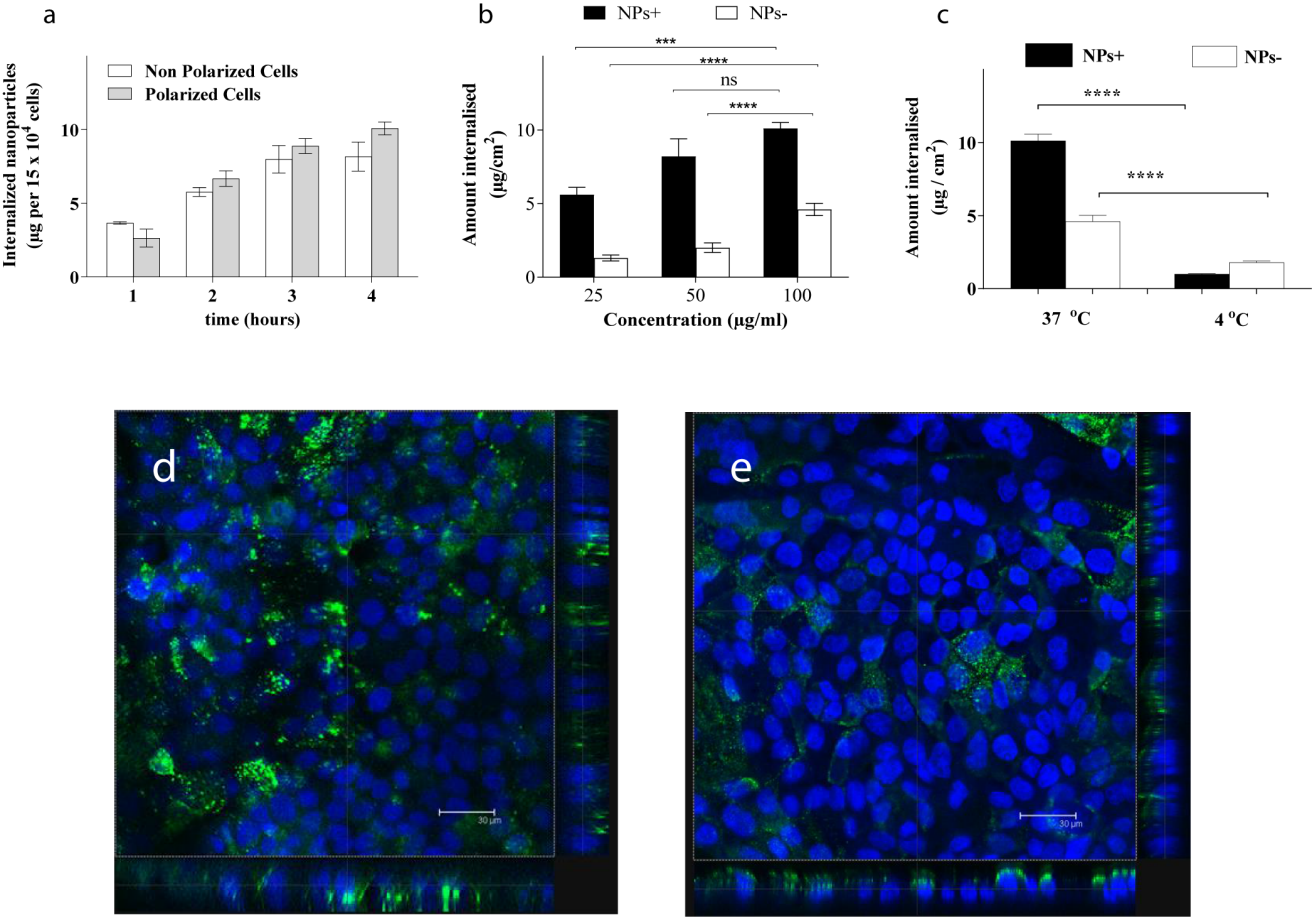
Internalization of nanoparticles by Caco-2 cells; (a) nanoparticle internalization by nonpolarized and polarized, differentiated Caco-2 cells; 100 μg/mL of 100 nm negatively charged nanoparticle (NP−) suspensions in HBSS:HEPES buffer applied; (b) effect of concentration and nanoparticle surface charge on cellular internalization; 100 nm positively charged nanoparticles (NPs+) and negatively charged nanoparticles (NPs−) suspended in HBSS:HEPES buffer applied at concentrations of 25, 50, and 100 μg/mL for 120 min; (c) internalization of nanoparticles at 4 and 37 °C. Data represent the mean ± SD (*n* = 4). *** and **** denote *p* < 0.001 and *p* < 0.0001, respectively, whereas “ns” indicates nonsignificant; (d) confocal microscopy overlay for Caco-2 cell layer incubated with ∼100 nm of positively charged nanoparticles at a concentration of 50 μg/mL; Hoechst 33 342-labeled cell nuclei are in blue; positively charged nanoparticles (labeled with a styryl pyran derivative fluorophore) are in green; scale bar is 30 μm; (e) confocal microscopy overlay for Caco-2 cell layer incubated with ∼100 nm of positively charged nanoparticles at a concentration of 50 μg/mL in the presence of dynasore inhibitor (80 μg/mL); Hoechst 33 342-labeled cell nuclei are in blue; positively charged nanoparticles (labeled with a styryl pyran derivative fluorophore) are in green; scale bar is 30 μm; edge panels in images d and e show x-z and y-z planes.

Prior to assessing cellular internalization pathways of nanoparticles, their toxicity to nonpolarized and polarized cells was assessed (Figure S3). The data for nonpolarized cells demonstrate increased toxicity of positively charged nanoparticles when applied concentration is increased above 100 μg/mL. Regarding the polarized cell layers, TEER profiles, taken as a measure of nanoparticle toxicity, which would decrease due to loss of layer integrity if cells experience toxic effects, also illustrate the negative effects of positively charged nanoparticles at concentrations above 100 μg/mL. Further studies of endocytosis were thus conducted using both sets of nanoparticles as 50 μg/mL suspensions. Figure 2 demonstrates a similar level of internalization of nanoparticles by nonpolarized and polarized cells at different exposure time points (graph a) and concentration-dependent, within the range tested, internalization of both positively and negatively charged nanoparticles by polarized cells, whereby a significantly higher, in mass of the nanoparticles per cell layer, internalization of positively charged particles, relative to negatively charged nanoparticles, is evident (graph b). In further studies, we normally applied 0.5 mL of 50 μg/mL of nanoparticle suspensions, and approximately 8% or 30% of applied nanoparticles was internalized by cells for negative and positive nanoparticles, respectively. The data obtained at 37 and 4 °C (graph c) illustrate a clear temperature dependence of nanoparticles internalization by cells, indicating that an active process is indeed responsible for the nanoparticle internalization.

Confocal images d and e in Figure 2 are illustrative of Caco-2 cell layers treated with positively charged nanoparticles in the absence or presence, respectively, of one of the used inhibitors, dynasore. It is evident from the edge panels in x*-*z and y*-*z planes in image d that the nanoparticles are present at the level of cell nuclei and through the depth of the planes, hence indicting their internalization by cells. It is furthermore evident that there is no “layer” of surface present - positively charged nanoparticles indicating that, if the nanoparticles had adsorbed to the surface of the cells during the treatment, these were removed by the washing protocol applied.^16^ The image further illustrates that internalization of the nanoparticles is not homogeneously distributed in the cell layer, in line with previous studies and our recent work.^17^ The image e illustrates that in the presence of dynasore, the nanoparticles are located predominantly above the cell nuclei level, toward the apical membrane, indicating that nanoparticles remain in the vicinity of the apical membrane, which would occur if dynasore’s presence prevents the nanoparticles from further intracellular trafficking.

### Internalization of Nanoparticles by Nonpolarized Cells in the Presence of Inhibitors

**Figure 3:**
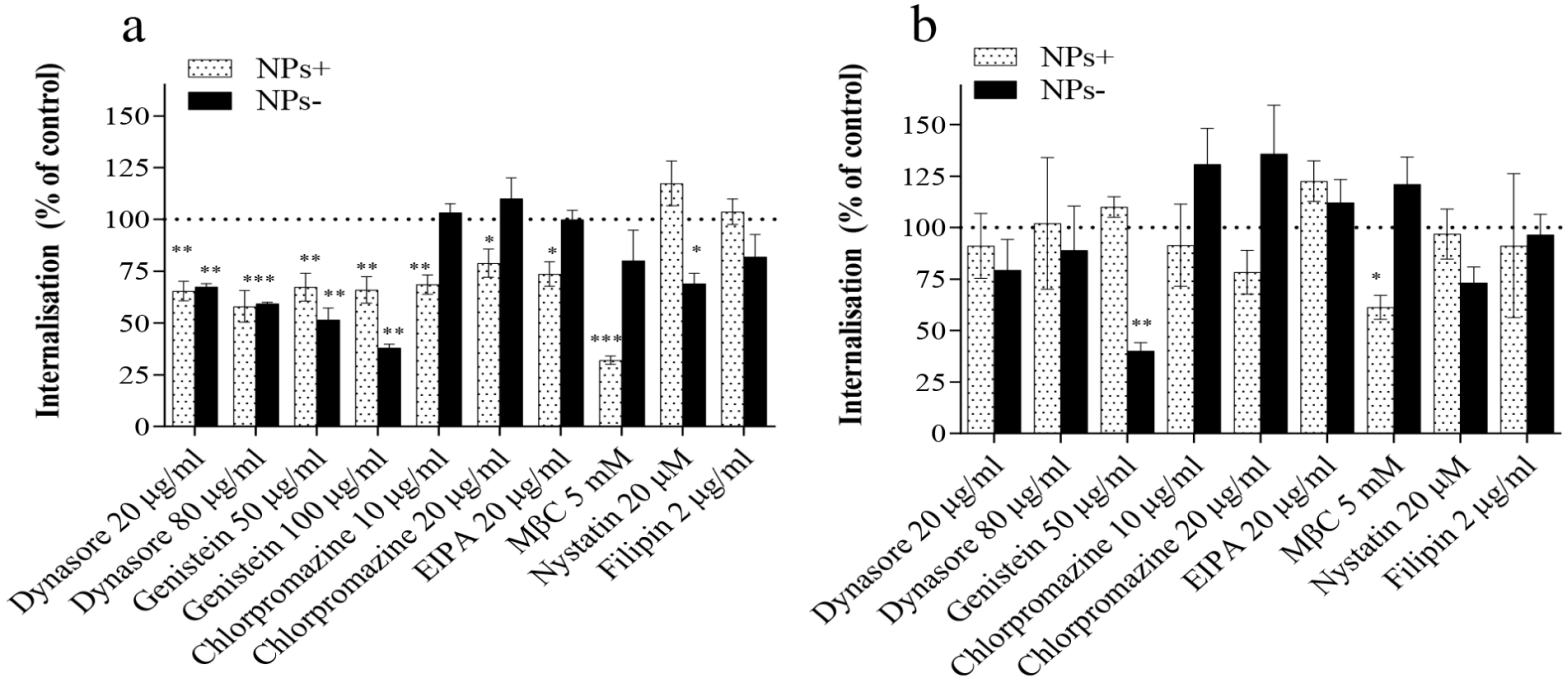
Cellular internalization of nanoparticles by nonpolarized, nondifferentiated Caco-2 cells in the presence of endocytosis inhibitors. Inhibitors were applied to nonpolarized Caco-2 cells 30 min prior to the application of nanoparticles (NPs), followed by (a) 120-min or (b) 180-min exposure to nanoparticles (at 50 μg/mL) in the presence of the same inhibitor. Results are reported as the amount of internalized nanoparticles relative to the control (cells not treated with an inhibitor), expressed as a percentage of the quantity found for the control cells at the same time point. Data represent the mean ± SD (*n* = 4). *, **, *** and **** indicate *p* < 0.05, *p* < 0.01, *p* < 0.001, and *p* < 0.0001, respectively, a statistically significant difference compared to control (untreated cells at the same time point). Relevant toxicity of inhibitors, their inhibition of transferrin and cholera-β-toxin, and effects of selected concentrations used on TEER of polarized cell layers are shown in Figures S5–S7. Concentrations of inhibitors applied are listed in Table S2.

Figure 3 summarizes data on cellular internalization of nanoparticles, applied in the presence of a panel of pharmacological inhibitors of endocytic pathways by nonpolarized Caco-2 cells. Data are expressed relative to the internalization of nanoparticles by cells, at the same time point but in absence of the inhibitors.

The main features of the data collected at 120 min exposure include: (i) a statistically highly significant reduction in cellular internalization of both negatively and positively charged nanoparticles in the presence of dynasore, the reversible inhibitor of dynamin-dependent, vesicular endocytosis; (ii) a significant reduction in internalization, and particularly prominent for negatively charged nanoparticles, in the presence of genistein, an inhibitor of protein tyrosine kinase (PTK), an enzyme that catalyzes tyrosine phosphorylation and activates downstream signal transduction, affecting multiple cellular function,^18^ among which it is implicated in lipid raft–dependent endocytosis, normally attributed to the caveolin pathway;^19^ (iii) a significant reduction in positively charged nanoparticle internalization in the presence of chlorpromazine, an inhibitor of clathrin-dependent pathway,^20,21^ and an absence of its effect for negatively charged nanoparticles; (iv) a significant reduction of positive nanoparticle internalization by EIPA, an agent generally understood to primarily inhibit macropinocytosis;^22,23^ (v) a reduction, and particularly significant for positive nanoparticles, of internalization by MβC, which sequesters cholesterol from cell membranes and can consequently alter the structure of cholesterol-rich domains (lipid rafts), which are implicated in the literature not only in caveolae-mediated endocytosis but also in clathrin-mediated pathway and macropinocytosis;^24,25^ and (vi) a reduction in the internalization of negatively charged nanoparticles by nystatin and filipin, cholesterol-binding inhibitors accepted to profoundly disrupt the structure and functions of cholesterol-rich membrane domains (lipid rafts) and particularly lead to aberrations in the shape of caveolae,^26^ and an absence of their effect on positively charged nanoparticles.

The inhibitor effects after 180 min of exposure appear somewhat subdued, i.e., extent of reduction in nanoparticle internalization seen at 120 min is less pronounced and is, in some cases, close to that of nontreated cells at the same time point. Studies on the time dependence of the effects of pharmacological inhibitors on endocytosis are scarce. The effects of some tested inhibitors were shown to be time dependent and reversible on their removal, for instance, dynasore,^27^ chlorpromazine,^28^ or nystatin, colchicine and amiloride;^29^ however, in this study, the experiments were conducted in the presence of inhibitors. Further studies are required in this area. It is, nevertheless, evident that significant reduction in the internalization persists at 180 min for negative nanoparticles by genistein (reported to be involved in lipid raft–dependent endocytosis) and for positive nanoparticles by MβC (cholesterol sequestering inhibitor), both indicating the important role of membrane-present cholesterol-dependent processes. The internalization of positive nanoparticles is further affected by the chlorpromazine inhibition of the clathrin pathway, although the effect is reduced relative to 120 min.

In general, the data point to (i) the significant role of dynamin-depended vesicular processes on nanoparticle internalization by nonpolarized cells, regardless of their surface charge, in addition to the profound effect of genistein—reported to be involved in lipid raft–dependent endocytosis but also shown to cause local disruption of the actin network at the site of endocytosis, which inhibits the recruitment of dynamin-2;^30^ (ii) profound “cholesterol dependence” of internalization, but with the effect conditional on the nanoparticle charge and nature of “cholesterol inhibition”; i.e., cholesterol depletion with MβC causes more dramatic decrease in the internalization of positively charged nanoparticles, with a modest effect on negatively charged nanoparticles, while cholesterol moderation by nystatin and fillip binding causes no apparent effects on positively charged nanoparticle but significantly impacts the internalization of negatively charged nanoparticles; and (iii) importance of clathrin as well as dynamin-independent macropinocytosis inhibited by EIPA in the internalization of positively charged nanoparticles.

### Internalization of Nanoparticles by Polarized Cells in the Presence of Inhibitors

**Figure 4:**
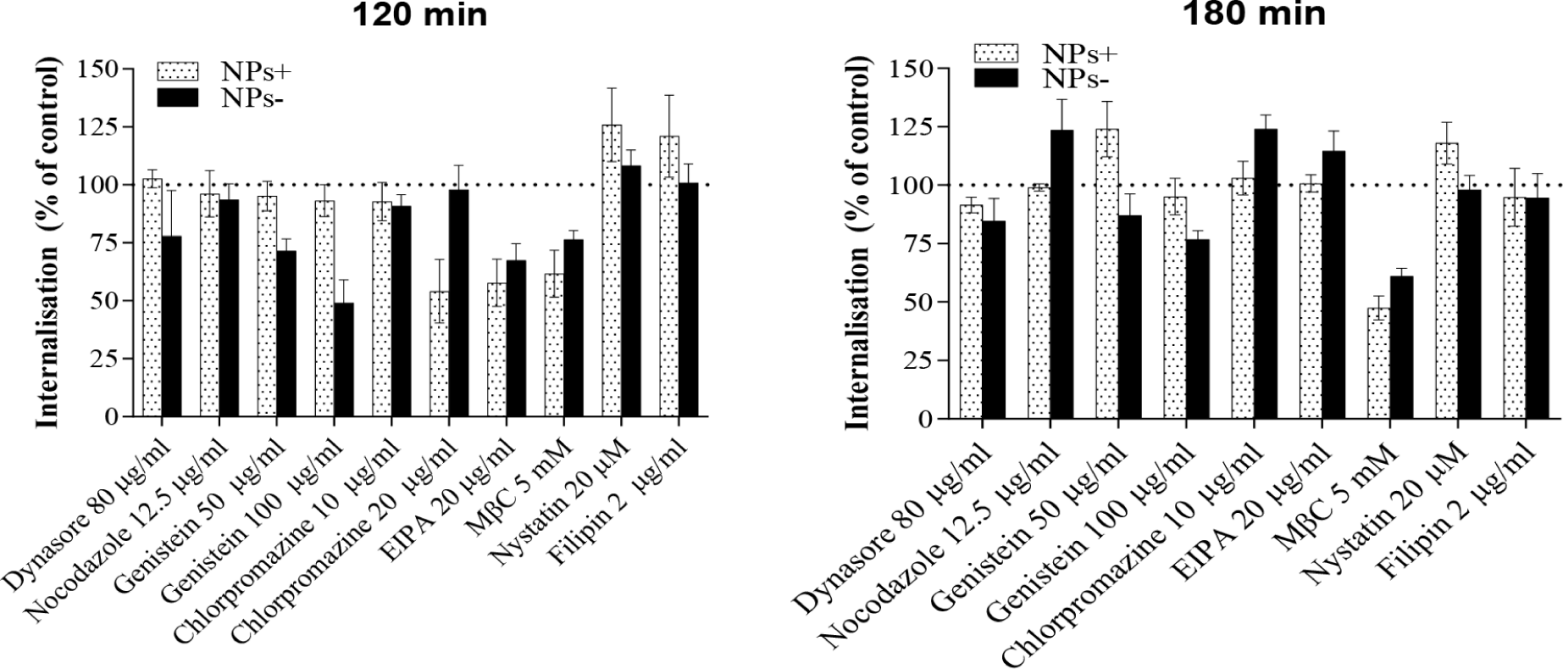
Internalization of nanoparticles by polarized, differentiated Caco-2 cell layers in the presence of endocytosis inhibitors. Inhibitors were applied to polarized Caco-2 cell layers 30 min prior to the application of nanoparticles (NPs), followed by (a) 120 min or (b) 180 min of exposure to nanoparticles (at 50 μg/mL) in the presence of the same inhibitor. Results are reported as the amount of internalized nanoparticles relative to the control (cell layers not treated with an inhibitor), expressed as a percentage of the quantity found for control cell layers (at the same time point). NPs+ denote positively charged nanoparticles and NPs– denotes negatively charged nanoparticles. Data represent the mean ± SD (*n* = 4). *, **, ***, and **** indicate *p* < 0.05, *p* < 0.01, *p* < 0.001, and *p* < 0.0001, respectively, a statistically significant difference compared to that of control (untreated cell layers).

Figure 4 summarizes nanoparticle internalization in the presence of endocytosis inhibitors by Caco-2 cells grown as polarized, differentiated cell layers. The data illustrate the following: (i) a moderate reduction in cellular internalization on dynasore inhibition of dynamin, and observed primarily for negatively charged nanoparticles, while dynamin-dependent internalization plays a pronounced role in nonpolarized cells for both positive and negative nanoparticles (Figure 3); (ii) a significant reduction in internalization in the presence of genistein for negatively charged nanoparticles; (iii) a significant reduction in internalization of positively charged nanoparticles in the presence of a higher concentration of chlorpromazine and an absence of its measurable effect on negatively charged nanoparticles; (iv) a significant reduction in internalization of both positive and negative nanoparticles on treatment with EIPA, the effect more prominent relative to nonpolarized cells; (v) a significant reduction in internalization of both sets of nanoparticles by MβC, and (vi) an absence of reduction in internalization of negatively charged nanoparticles by nystatin and filipin, seen in nonpolarized cells.

In polarized cell layers, we further tested the role of microtubules on nanoparticle internalization applying nocodazole, a microtubule-depolymerizing agent,^31^ as a previous study^32^ indicated a significant inhibition in cell internalization of positively charged nanoparticles but displayed no effect on the internalization of negatively charged nanoparticles of similar size. The results appear to indicate an insignificant effect of this inhibition on the cellular internalization. This, however, does not preclude microtubule involvement in subsequent cellular transport.^11^

At 180 min, the extent of inhibition by different inhibitors is reduced, similar to the time-effect seen in nonpolarized cells (Figure 2). The effects of dynasore and genistein inhibitions on negatively charged nanoparticle internalization are now moderate, while MβC’s cholesterol depletion is still showing a pronounced inhibition of both positive and negative nanoparticle internalization. Of interest, a significant inhibition of nanoparticle internalization by EIPA seen at 120 min is reduced, for both positively charged and negatively charged nanoparticles.

**Figure 5:**
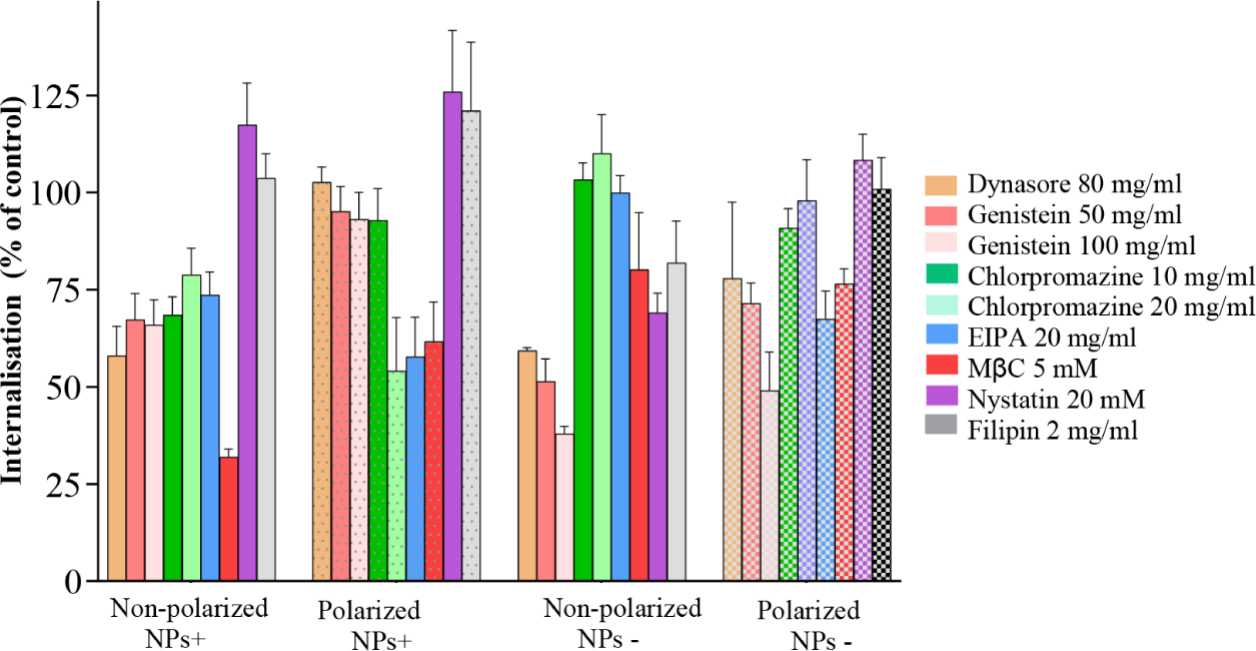
Comparison of nanoparticle internalization in nonpolarized and polarized Caco-2 cells; to assist comparisons, data at 120 min from Figures 3 and 4 are summarized.

Comparing data for cellular internalization in nonpolarized and polarized cells (as summarized in Figure 5), few features are evident: (i) significant dynasore inhibition observed in nonpolarized cells, for both positively charged and negatively charged nanoparticles, is reduced in polarized cells, particularly significantly for positively charged nanoparticles; (ii) prominent EIPA’s effect at 120 min in polarized cells is in contrast to its moderate inhibition of internalization of positively charged nanoparticles and no significant influence on negatively charged nanoparticles in nonpolarized cells—indicating a prominent role of macropinocytosis in polarized cells, the pathway that is considered dynamin independent and is distinct from clathrin- and caveolae-mediated pathways; (iii) reduced effect of cholesterol-binding inhibitors on the internalization of negatively charged nanoparticles in polarized, relative to nonpolarized cells.

Regarding dynamin-dependent endocytosis, dynasore inhibition works by blocking the enzymatic activity of dynamin, a GTP-dependent enzyme that self-assembles into a helical collar around the neck of newly formed cell membrane invagination and aiding their fission from the cell membrane to form intracellular vesicles.^33^ In polarized cells, dynamin further plays an important role in epithelial cell polarization, being a central player in the assembly and maintenance of intercellular junctions^34^ and playing other distinct regulatory roles, including its function in membrane trafficking; hence, its inhibition would be expected to show a profound impact on *transport* across epithelial cell layers, as we previously observed.^35^ Previous studies employing dynasore inhibition reported variable results, whereby a significant effect in polarized Caco-2 layer was reported for endocytosis of different cargos, e.g., Echovirus 1 virus,^36^ dendrimers,^37^ or solid lipid nanoparticles.^38^ Of interest, it was described in HeLa cells, human U373-MG (astrocytes), and monkey COS-1 (fibroblast-type) and BSC-1 (epithelial kidney) cell lines that dynasore inhibition of endocytosis is significant at a cell density of 40 to 70%, while denser cultures were reported more resistant to the effects of dynasore.^27^ It was concluded that dynamin expression and phenotype were highly dependent on the confluency of the cells.

Considering a prominent impact of EIPA, i.e., a pronounced inhibition of internalization for both positive and negative nanoparticles in the polarized cell layer, this indicates a prominent role of macropinocytosis in polarized cells, contrary to nonpolarized cells. Macropinocytosis is defined by actin-mediated cell membrane ruffling, occurring transiently due to, e.g., growth-factor induced actin polymerization at the cell surface, ultimately leading to macro-pinosome formation.^39^ In the light of differences of actin organization in nonpolarized and polarized cells, whereby in polarized cell layers a horizontal network underlying the apical surface is present in which actin organizes to form apical microvilli^5^ (as seen in Figure 1), it appears that the observed prominent role of EIPA reflects this characteristic of polarized cells. It is, however, not clear at this stage what may be the specific stimulation factor/s involved in nanoparticle-induced macropinocytosis^39^ in our study. It is important to note that the acidification of the cytosol (due to EIPA inhibition of the Na^+^/H^–^ exchange located in membrane) does not affect the formation of clathrin-coated pits but that it can affect the later stages of clathrin-mediated endocytosis since budding off of clathrin-coated pits from the membrane seems to be affected.^40^

Cholesterol depletion from cell membrane by MβC shows pronounced inhibition of internalization of nanoparticles in both cell cultures, i.e, nonpolarized and polarized cells (Figures 3 and 4). A difference should be noted here, whereby the apical membrane in polarized cells is shown to be enriched with cholesterol and glycosphingolipids species,^7^ designed to maintain its structural rigidity and reduce apical permeability. Cholesterol plays a multifaceted role in the cell membrane; it is an essential component for the formation of cell membrane lipid rafts, caveolae structures are rich in cholesterol,^41^ it is important for the clathrin-mediated pathway, and it also plays a role in macropinocytosis,^25^ as seen, for instance, in the uptake of DNA lipoplexes.^42^ The latter is also important to consider in the present study as it demonstrates that macropinocytosis plays an important role in nanoparticle internalization in differentiated cell layers (Figure 4), which would be hence affected by cholesterol depletion. However, with the “promiscuous” role of cholesterol in multiple cell membrane and endocytosis processes, cholesterol depletion, or its modulation, does not allow a distinction between endocytic mechanisms.

Concerning cholesterol modulation by binding agents nystatin and filipin, shown to affect lipid raft–dependent endocytic processes^43-^—often ascribed to caveolae-mediated endocytosis, these agents show no effect on the internalization of positive nanoparticles in both nonpolarized and polarized cells, while their inhibition of negatively charged nanoparticle internalization in nonpolarized cells is not seen in polarized cell layers. Relevant to the latter observation and considering a possible role of cholesterol-rich caveolae structures in endocytosis by Caco-2 cells, there is a lack of consensus in the literature on expression, and cellular location, of caveolin-1, a protein essential in the formation of caveolae, in these cells. A number of studies reported its expression and its role in endocytosis and in apical to basolateral transport,^44,45^ with expression shown at mRNA as well as protein level (by immuno-fluorescence) at the apical membrane and within the cytoplasm of differentiated Caco-2 cells. Caveolae-mediated entry of a number of viruses into Caco-2 cells at the apical membrane has been reported, including recently the internalization and transport of SARS-CoV-2.^1^ On the other side are publications reporting the absence of caveolin expression and endocytic role in Caco-2 cells.^46,47^ We have, in our previous study,^48^ as well as here, observed the expression of caveolin-1 in polarized Caco-2 cells at mRNA level and by antibody immunostaining; however, permeabilization of cells to conduct the immunoassay, and microscopy applied, does not allow us to clearly ascertain the protein’s cellular location at the apical plasma membrane (x-z and y-z planes in Figures 1 and S4). Intriguingly, a significant impact of genistein, usually assigned a role of an inhibitor of caveolae-mediated cell entry,^19^ is observed on the endocytosis of negatively charged nanoparticles in both nonpolarized cells and polarized cells, while the endocytosis inhibition seen in nonpolarized cells by cholesterol-binding nystatin and filipin, also assigned to caveolae-mediated endocytosis, is not present in polarized cells. Genistein is a general tyrosine kinase inhibitor^49^ that works by preventing actin depolymerization in the local cytoskeleton, which precedes the internalization of endocytic vesicles, and by preventing the recruitment of dynamin-2 required for the scission of vesicles from cell membrane and hence has been shown to impact on other endocytosis processes.^50^

This study contributes to an extensive literature on endocytosis, particularly to a growing body of studies on the role of cellular polarization on the endocytosis processes, all demonstrating complexity in the roles and interplay of cell membrane structures and its lipid and protein organization.^51^ The work here also points to the role of cargo endocytosed, however “passive” it may be, in determining the endocytic process involved.

## Conclusions

This study compares cellular internalization of nanoparticles in Caco-2 epithelial cells grown as either nonpolarized or polarized differentiated cells. Our data collectively indicate that endocytosis pathways are affected by both the state of cell differentiation (i.e nonpolarized versus differentiated, polarized cells) as well as the nature of cargo endocytosed (positive versus negative nanosized particles). In polarized cells, actin-driven and dynamin-independent macropinocytosis plays a prominent role in the internalization of both positively and negatively charged cargo, contrary to its moderate contribution in nonpolarized cells. In polarized cells, clathrin plays a prominent role in the endocytosis of positively charged nanoparticles and cholesterol inhibition by genistein in negatively charged nanoparticles. In nonpolarized cells, dynamin-dependent endocytosis is prevailing in the internalization of both positively charged and negatively charged nanoparticles, while there is only modest contribution of macropinocytosis in the internalization of positively charged nanoparticles. Cholesterol depletion by methyl-ß-cyclodextrin plays a prominent role in both nonpolarized and polarized cells for the internalization of both positively charged and negatively charged nanoparticles, in addition to the effect of cholesterol-binding inhibitors on the internalization of negatively charged nanoparticles in both cell cultures, all indicating the multifaceted roles of plasma membrane–associated cholesterol.

## Supporting Information Available

Nanoparticle characterization and toxicity, toxicity of pharmacological inhibitors, and effect on internalization and “classical” pathway ligands (PDF)
