# Supplementary material for: Difference in Endocytosis Pathways Used by Differentiated Versus Nondifferentiated Epithelial Caco-2 Cells to Internalize Nanosized Particles
Source: Mol Pharm. 2024 Jun 12;21(7):3603–12. doi: 10.1021/acs.molpharmaceut.4c00333 (PMC11220748; doi:10.1021/acs.molpharmaceut.4c00333)
Supplement: Supplementary file 1 — mp4c00333_si_001.pdf [file mp4c00333_si_001.pdf]

Supporting information

On the difference in endocytosis pathways used by differentiated *versus* non-differentiated epithelial Caco-2 cells to internalise nano-sized particles

Azzah Bannunah[a], Robert Cavanagh[a], Saif Shubber[a], Driton Vllasaliu[b] and Snow Stolnik[a*]

[a] School of Pharmacy, University of Nottingham, University Park, Nottingham NG7 2RD, UK

[b] School of Cancer & Pharmaceutical Sciences, Faculty of Life Sciences & Medicine, King's College London, Franklin-Wilkins Building, 150 Stamford Street, London SE1 9NH, UK

* corresponding author

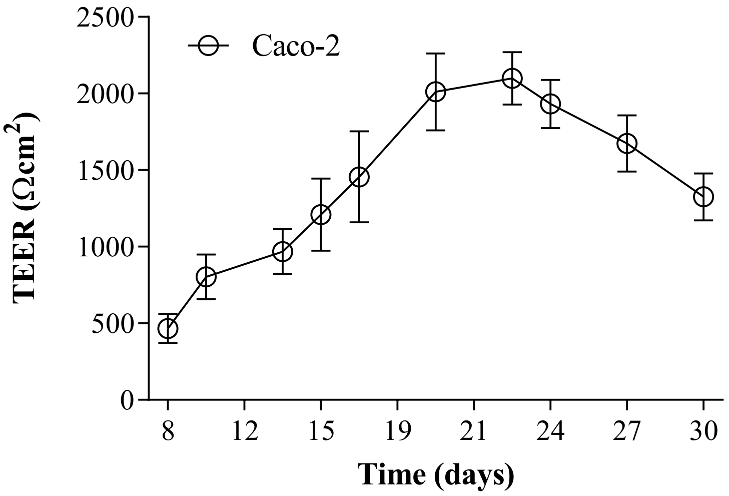

Figure S1. *TEER profile of Caco-2 cells cultured on permeable inserts. Background TEER due to the permeable membrane inserts was subtracted from the reported TEER values. Data are presented as the mean ± SD (n = 10 to12).*

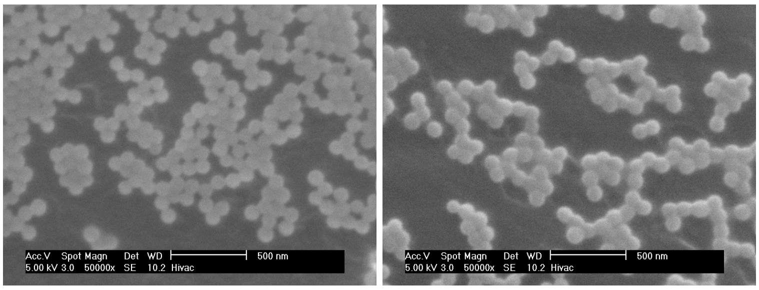

Figure S2. *Scanning electron microscopy images of polystyrene nanoparticles used in the study.*

Table S1. *Dynamic Light Scattering measurements of the hydrodynamic radius (nm) of positively and negatively charged nanoparticles in the biological medium, HBSS:HEPES at pH 7.4. Values represent the mean of 10 measurements at 25°C. * Nominal size given by suppliers used in the text.*

**Nanoparticles**	**Hydrodynamic radius (nm)**	**Zeta potential (mV)**
‘Positive 100 nm’	65.0 ± 3.5	+ 26.3 ± 2.3
‘Negative 100 nm’	45.5 ± 5.0	- 32.1 ± 1.7

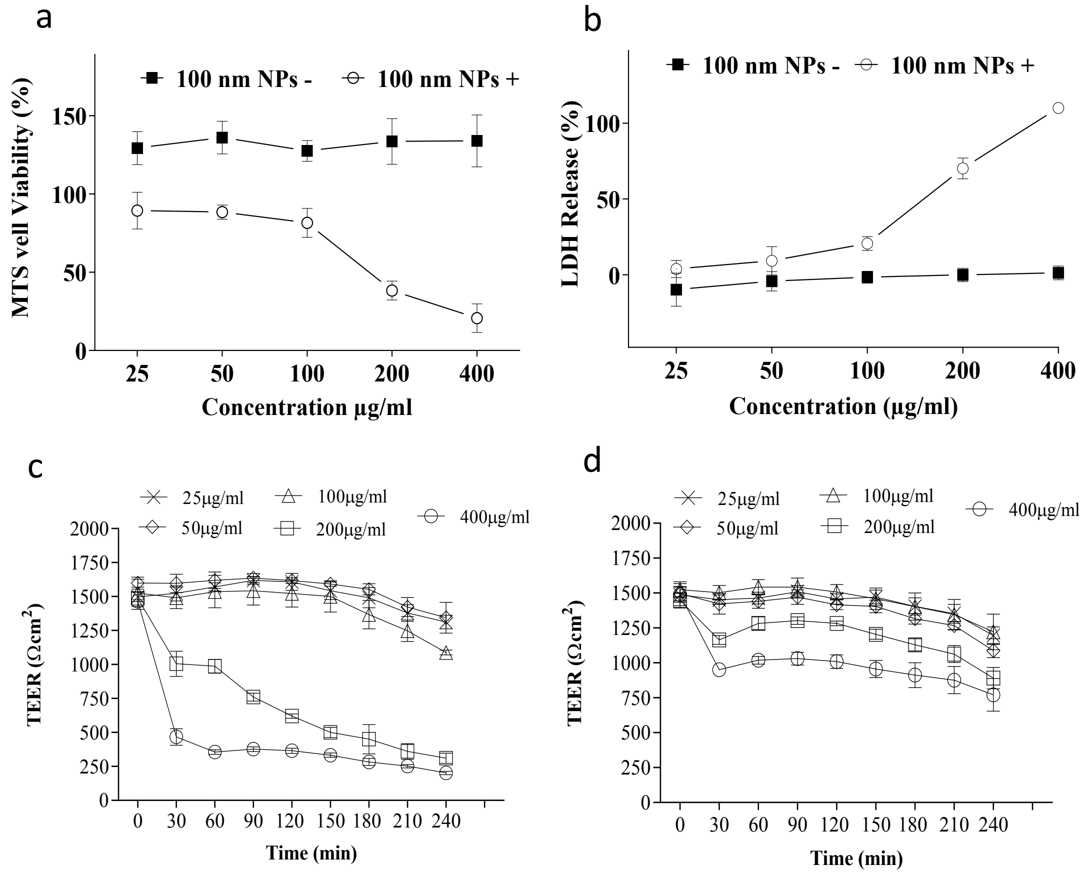

Figure S3. *Toxicity profiles of nanoparticles applied to Caco- 2 cells. a) and b) non-differentiated cells grown as standard culture on plasticware; effect of varying concentrations of positively (NPs+) and negatively (NPs−) charged nanoparticles on Caco-2 metabolic activity assessed by a) MTS (CellTiter 96® AQueous MTS) and b) LDH (Sigma TOX7, UK) assays; both assays performed as per suppliers' instructions. c) and d) differentiated cell layer grown on semipermeable Transwell® membrane; effect of varying concentrations of nanoparticles on TEER profiles for c) positively (NPs+) and d) negatively (NPs−) charged nanoparticles; nanoparticles were applied to the apical side of a Caco-2 layer, the layers were pre-incubated for 45 min with HBSS:HEPES, followed by 240 min (4 hours) of nanoparticle exposure at apical concentrations of 25, 50, 100, 200 and 400 µg/ml. TEER was measured every 30 min over the 240 min period.*

TEER is widely taken as a measure for toxicity of cell layer, as reviewed in Srinivasan, Balaji et al. "TEER measurement techniques for in vitro barrier model systems." Journal of laboratory automation vol. 20,2 (2015): 107-26. doi:10.1177/2211068214561025

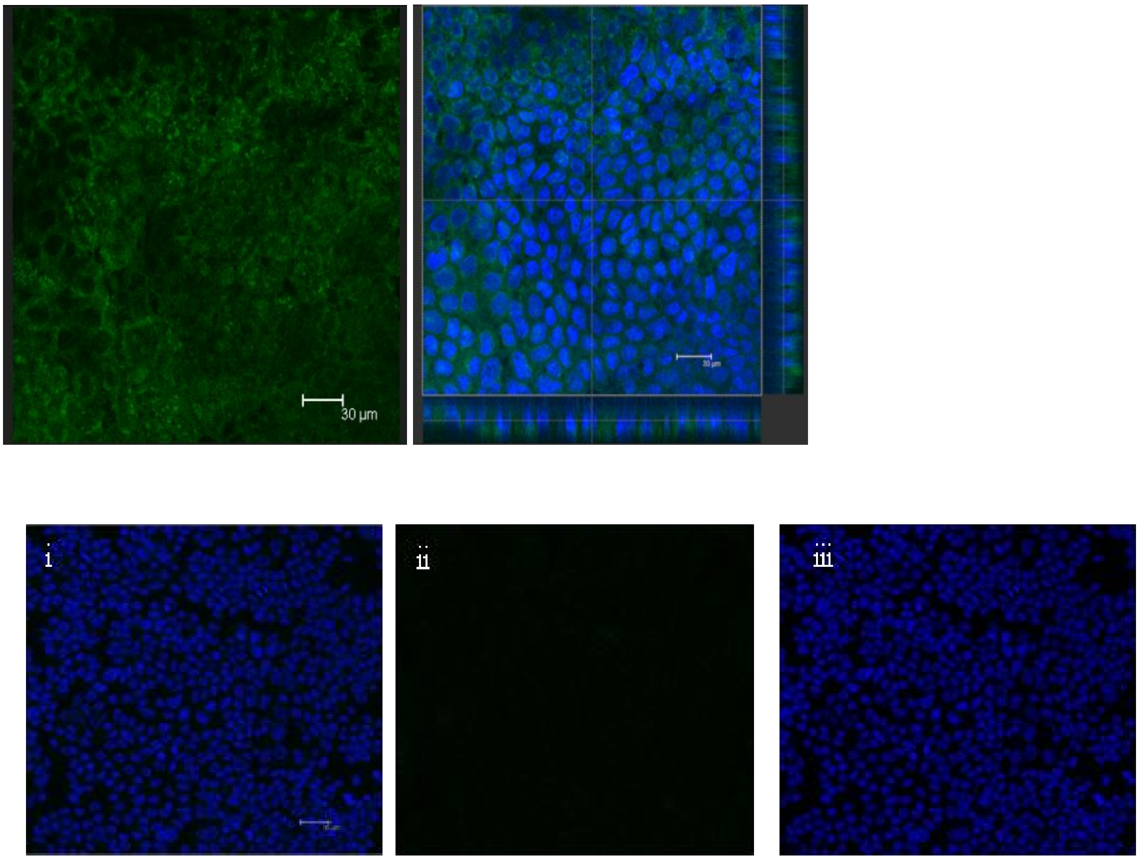

Figure S4. *Top row images: Expression of caveolin-1 in differentiated Caco-2 cells by immunostaining; cell layers permeabilised (as described in Materials and Methods) and treated with anti-human caveolin-1 H-97 antibody, followed by anti-rabbit IgG-FITC and overlay of cell layers, respectively, green is cavolin-1 immunostaining and blue is Hoechst-labelled cell nuclei.*

Bottom row images i-iii: show the negative control where no primary anti-body was applied, (i) Hoechst-labelled cell nuclei, (ii) cells treated with only secondary antibody, goat, anti-rabbit IgG-FITC, and (iii) overlay of cell layers.

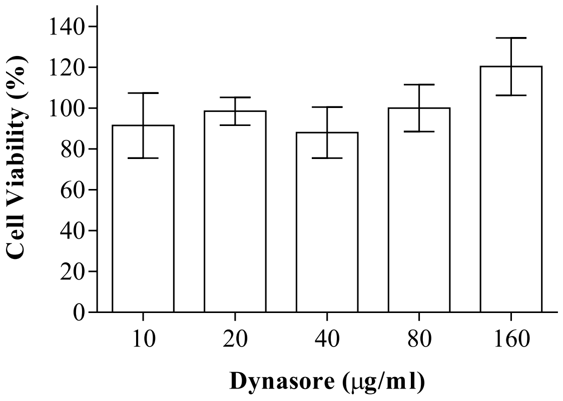
Cell Viability (%)
0
20
40
60
80
100
120
140
10
20
40
80
160
Dynasore (μg/ml)

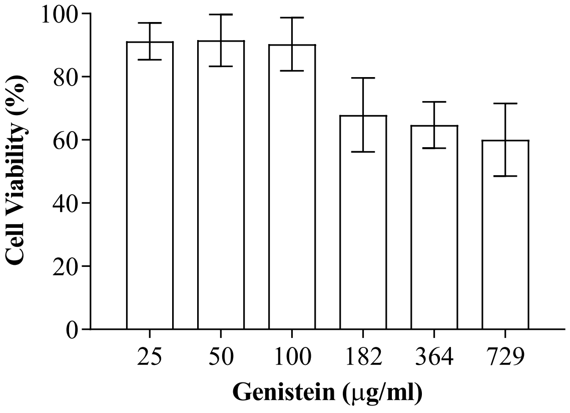
Cell Viability (%)
0
20
40
60
80
100
25
50
100
182
364
729
Genistein (μg/ml)

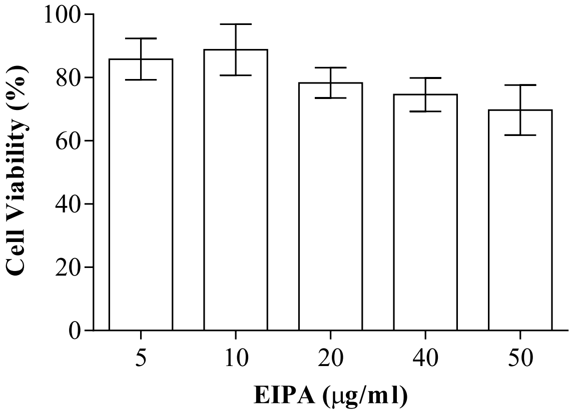
Cell Viability (%)
0
20
40
60
80
100
5
10
20
40
50
EIPA (μg/ml)

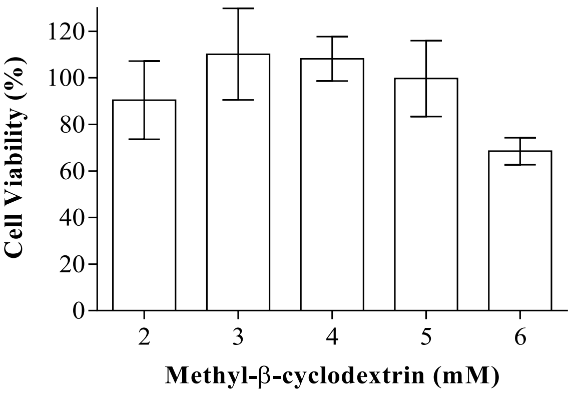
Cell Viability (%)
0
20
40
60
80
100
120
2
3
4
5
6
Methyl-β-cyclodextrin (mM)

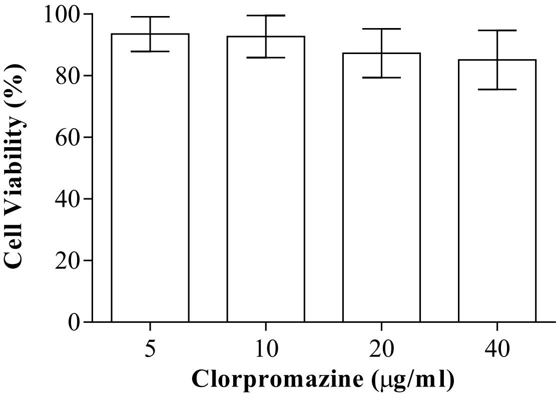
Cell Viability (%)
0
20
40
60
80
100
5
10
20
40
Clorpromazine (μg/ml)

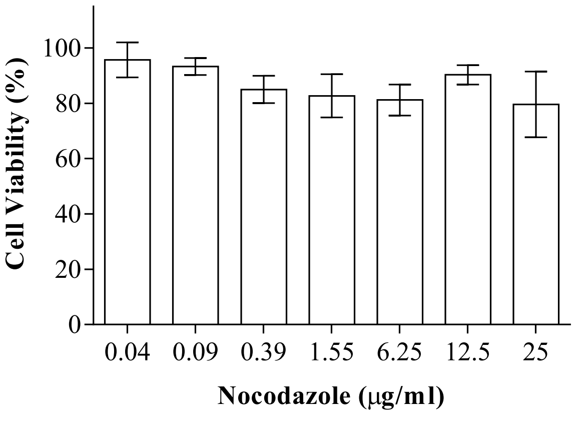
Cell Viability (%)
0
20
40
60
80
100
0.04
0.09
0.39
1.55
6.25
12.5
25
Nocodazole (μg/ml)

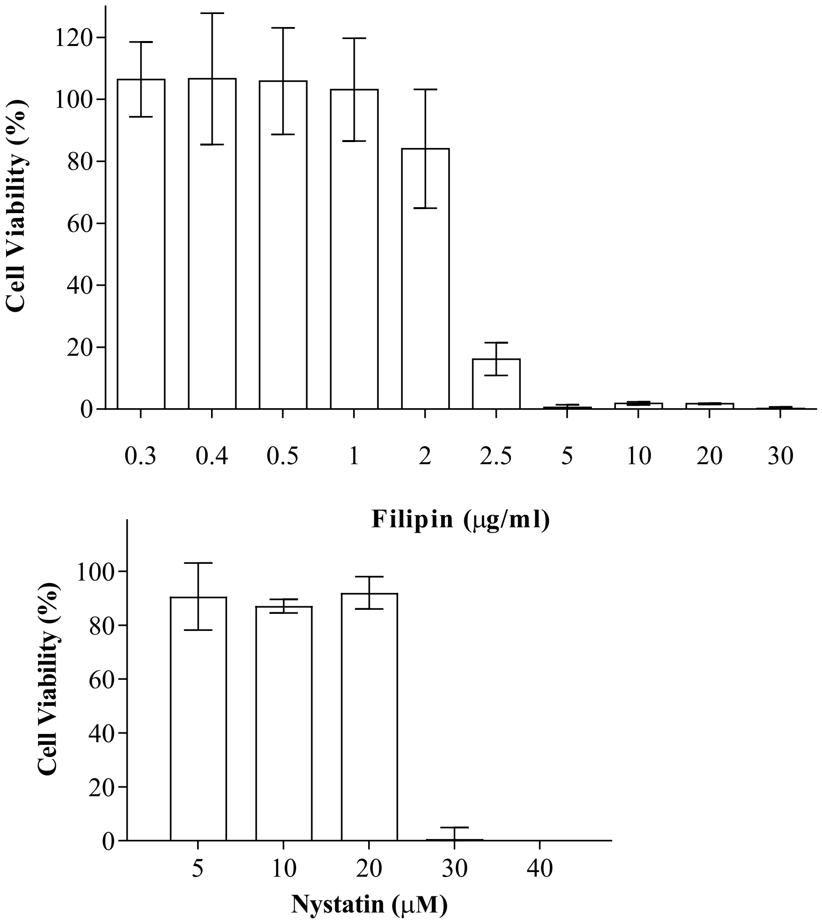

Figure S5. *Relative cell viability (%) after incubation of Caco-2 cells with different endocytosis inhibitors for 4 hours. Data are the results of an MTS assay; they are expressed as relative cell metabolic activity and presented as the mean ± SD (n =7).*

Table S2. *Selected concentrations of inhibitors and their recognised effect on cell internalization pathways*

Inhibitors	Concentrations	Cell viability (%)	Pathway & mechanism	
Dynasore	80 µg/ml	98	Clathrin and caveolae endocytosis; Inhibitor of dynamin, prevents scission of endocytic vesicles	[1]
Chlorpromazine	10 and 20 µg/ml	<87	Clathrin endocytosis; causes translocation of clathrin from membrane to intracellular vesicles	[2]
EIPA	10 µg/ml	88	Macropinocytosis; inhibits Na^+/H^- exchange	[3]
Nystatin	20 µM	92	Caveolae endocytosis; sequesters membrane cholesterol	[4]
Genistein	50 and 100 µg/ml	<90	Caveolae endocytosis; Tyrosine kinase inhibitor	[5]
Filipin	2 µg/ml	84	Caveolae endocytosis; Binds to membrane cholesterol	[6]
MβC	5 mM	99.6	Lipid raft endocytosis / Caveolae / Macropinocytosis; Depletes membrane cholesterol	[7]

[1] T. Kirchhausen, E. Macia, H.E. Pelish, Use of dynasore, the small molecule inhibitor of dynamin, in the regulation of endocytosis., Methods Enzymol. 438 (2008) 77–93.

[2] Z.M. Qian, H. Li, H. Sun, K. Ho, Targeted drug delivery via the transferrin receptor-mediated endocytosis pathway. Pharmacol. Rev. 54 (2002) 561–87.

[3] Gekle, M., Drumm, K., Mildenberger, S., Freudinger, R., Gassner, B. and Silbernagl, S. (1999) Inhibition of Na+-H+ exchange impairs receptor-mediated albumin endocytosis in renal proximal tubule-derived epithelial cells from opossum. Journal of Physiology-London. 520, p. 709-721.

[4] Y. Chen, S. Wang, X. Lu, H. Zhang, Y. Fu, Y. Luo, Cholesterol sequestration by nystatin enhances the uptake and activity of endostatin in endothelium via regulating distinct endocytic pathways., Blood. 117 (2011) 6392–403.

[5] T. Akiyama, J. Ishida, S. Nakagawa, H. Ogawara, S. Watanabe, N. Itoh, et al., Genistein, a specific inhibitor of tyrosine-specific protein kinases., J. Biol. Chem. 262 (1987) 5592–5.

[6] J.E. Schnitzer, P. Oh, E. Pinney, J. Allard, Filipin-sensitive caveolae-mediated transport in endothelium: reduced transcytosis, scavenger endocytosis, and capillary permeability of select macromolecules., J. Cell Biol. 127 (1994) 1217–32.

[7] A. Christian, M. Haynes, M. Phillips, G. Rothblat, Use of cyclodextrins for manipulating cellular cholesterol content, J. Lipid Res. 38 (1997) 2264–2272.

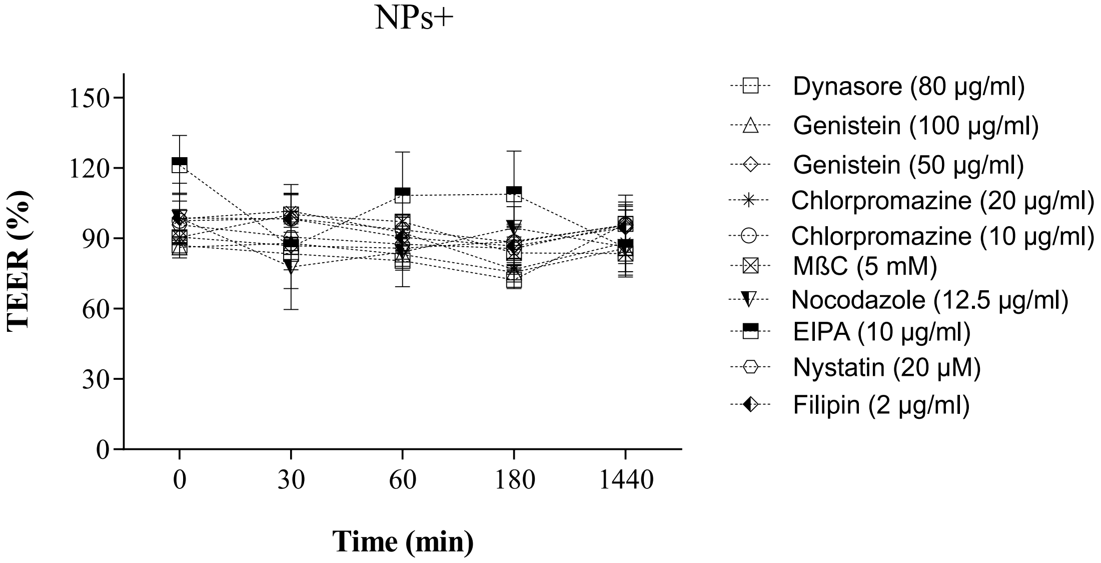

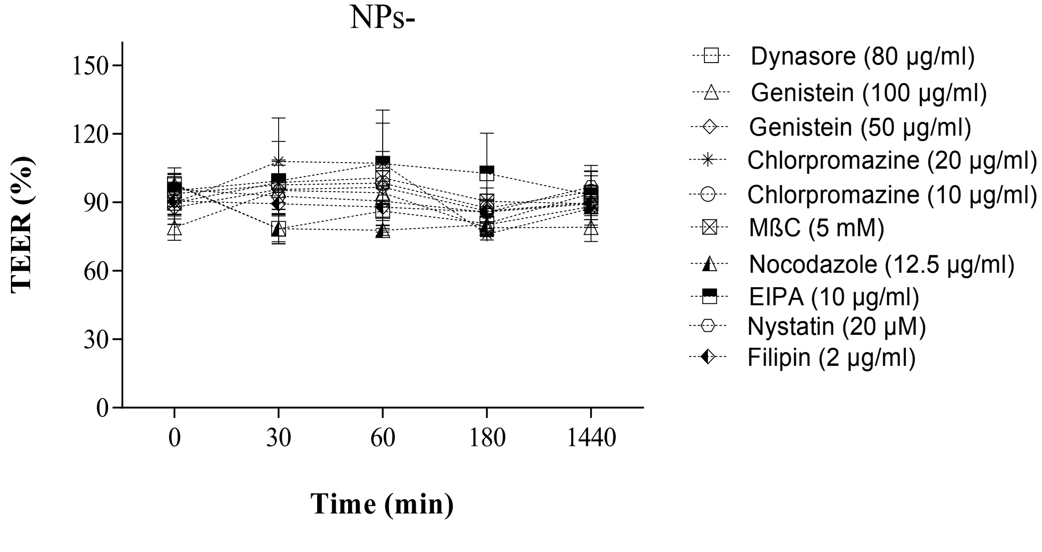

Figure S6. *The effect of endocytosis inhibitors in nanoparticles internalization experiment on the transepithelial electrical resistance (TEER) of Caco-2 cell layers. Cell layers were pre-incubated for 30 minutes with the inhibitors at the apical side, followed by 180 minutes of exposure to the nanoparticles (50 µg/ml), in the presence of the same inhibitors. TEER was measured every 30 minutes over 180 minutes, followed by sample removal and addition of the culture medium. After incubation for 1440 minutes (24 hours) in normal cell culture conditions (37°C), TEER was measured again to determine the recovery. Results are displayed as a % of the control (inhibitor-free) TEER value. Data represent the mean ± SD (n = 4).*

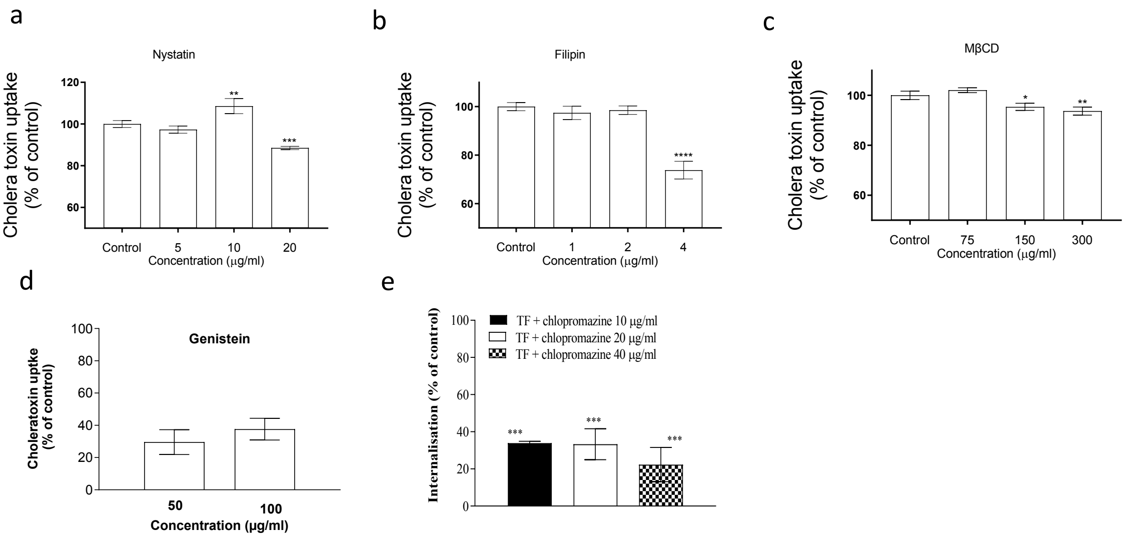

Figure S7. The effect of pharmacological inhibitors on cholera-β-toxin (CβT) transferrin (TF) uptake in non-polarized cells. *a) – d) nystatin, filipin, MβCD and genistein effect on internalisation of cholera-β-toxin in non-polarised Caco-2 cells. Cells were pre-treated with the inhibitors for 30 min, followed by 180 min of exposure to Alexa fluor labelled cholera-β-toxin (CβT) in the presence of the respective inhibitor; e) effect of chlorpromazine on internalisation of transferrin (TF), cells were pre-treated with the inhibitor for 30 min, followed by 180 min of exposure to Alexa fluor labelled transferrin in the presence of the inhibitor. Uptake is expressed as % of the control (inhibitor-free) value. *** indicates $p<0.001$ a statistically difference compared to control (untreated cell layers).*

Comments:

In this study a treatment with filipin at non-toxic concentration of 2 µg/ml was not efficient in inhibiting caveolae-mediated endocytosis of cholera-β-toxin. It has been reported that treatment of Caco-2 cells with 5 µg/ml filipin can reduce the uptake of cholera-βtoxin by about 20% (DOI: 10.1242/jcs.114.20.3737), but in our study this concentration showed a significant cell toxicity. Despite this observation, we proceeded with filipin treatment in the view of reported complexity and different observations of cholera-β-toxin pathways of cellular internalisation in different cells (DOI: 10.1242/jcs.114.20.3737), in addition to using another cholesterol affecting compound, nystatin, in our study.

Regarding macropinocytosis, there is an unanswered question of usefulness of fluid-phase 'markers', as discussed recently (DOI: 10.3390/membranes10080177).

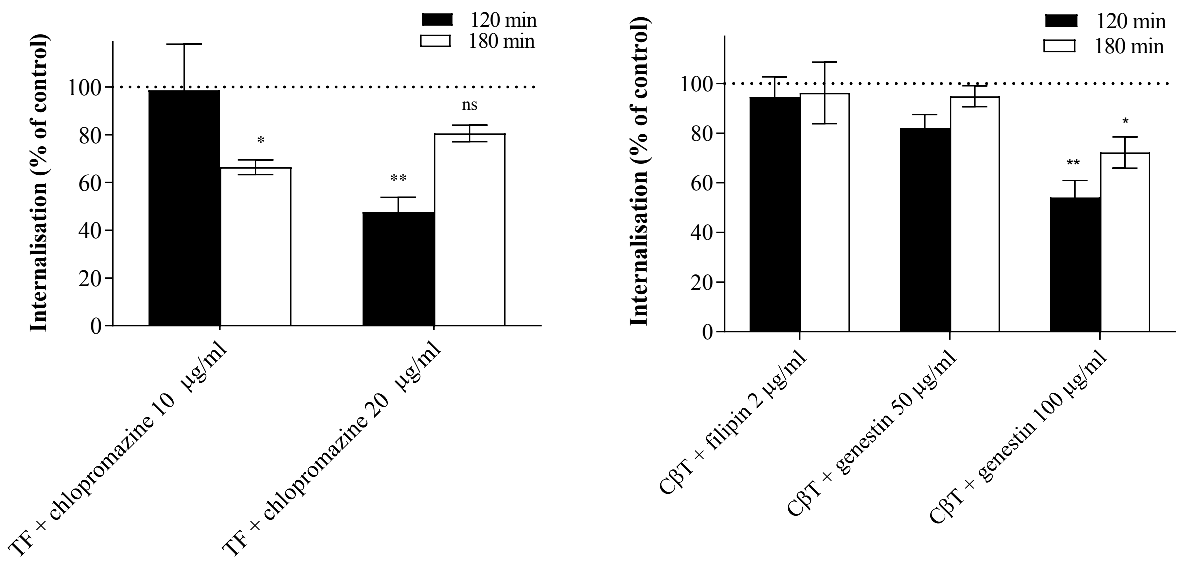

Figure S8: The effect of pharmacological inhibitors on internalisation of transferrin (TF) and cholera-β-toxin (CβT) in polarized Caco-2 cell layers. *The cell layers were pre-treated with an inhibitor solution for 30 min, followed by 120 min and 180 min of exposure to Alexa fluor labelled transferrin or Alexa fluor labelled cholera-β-toxin (CβT) in the presence of the inhibitor. * and ** Indicate $p<0.05$ and $p<0.01$, respectively, a statistically difference compared to control (untreated cell layers) whereas ns indicates non-significant.*
